# Characterization of the serum metabolome following radiation treatment in patients with high-grade gliomas

**DOI:** 10.1186/s13014-016-0626-6

**Published:** 2016-04-02

**Authors:** Lina Mörén, Carl Wibom, Per Bergström, Mikael Johansson, Henrik Antti, A. Tommy Bergenheim

**Affiliations:** Department of Chemistry, Computational Life Science Cluster, Umeå University, SE 901 87 Umeå, Sweden; Department of Radiation Sciences, Oncology, Umeå University, SE 901 85 Umeå, Sweden; Department of Clinical Neuroscience, Neurosurgery, Umeå University, SE 901 85 Umeå, Sweden; Department of Chemistry, Umeå University, SE 90187 Umeå, Sweden

**Keywords:** Glioblastoma, Radiation therapy, Treatment response, Metabolomics, Chemometrics

## Abstract

**Background:**

Glioblastomas progress rapidly making response evaluation using MRI insufficient since treatment effects are not detectable until months after initiation of treatment. Thus, there is a strong need for supplementary biomarkers that could provide reliable and early assessment of treatment efficacy. Analysis of alterations in the metabolome may be a source for identification of new biomarker patterns harboring predictive information. Ideally, the biomarkers should be found within an easily accessible compartment such as the blood.

**Method:**

Using gas-chromatographic- time-of-flight-mass spectroscopy we have analyzed serum samples from 11 patients with glioblastoma during the initial phase of radiotherapy. Fasting serum samples were collected at admittance, on the same day as, but before first treatment and in the morning after the second and fifth dose of radiation. The acquired data was analyzed and evaluated by chemometrics based bioinformatics methods. Our findings were compared and discussed in relation to previous data from microdialysis in tumor tissue, i.e. the extracellular compartment, from the same patients.

**Results:**

We found a significant change in metabolite pattern in serum comparing samples taken before radiotherapy to samples taken during early radiotherapy. In all, 68 metabolites were lowered in concentration following treatment while 16 metabolites were elevated in concentration. All detected and identified amino acids and fatty acids together with myo-inositol, creatinine, and urea were among the metabolites that decreased in concentration during treatment, while citric acid was among the metabolites that increased in concentration. Furthermore, when comparing results from the serum analysis with findings in tumor extracellular fluid we found a common change in metabolite patterns in both compartments on an individual patient level. On an individual metabolite level similar changes in ornithine, tyrosine and urea were detected. However, in serum, glutamine and glutamate were lowered after treatment while being elevated in the tumor extracellular fluid.

**Conclusion:**

Cross-validated multivariate statistical models verified that the serum metabolome was significantly changed in relation to radiation in a similar pattern to earlier findings in tumor tissue. However, all individual changes in tissue did not translate into changes in serum. Our study indicates that serum metabolomics could be of value to investigate as a potential marker for assessing early response to radiotherapy in malignant glioma.

**Electronic supplementary material:**

The online version of this article (doi:10.1186/s13014-016-0626-6) contains supplementary material, which is available to authorized users.

## Background

High-grade gliomas constitute a challenge for the treating physician. Despite large efforts when treating these tumors the median survival is only approximately 14 months at best [1]. Today the standard treatment is based on surgery followed by radiochemotherapy and adjuvant temozolomide. When these measures fail, surgery may be considered as well as second line chemotherapy and re-irradiation in selected cases.

New treatment strategies are needed for malignant gliomas and there is currently no lack of new candidate targets. However, one of the major problems when treating those tumors is the lack of immediate monitoring of the treatment effects. The tumors are fast growing and response evaluation with repeated CT or MRI is not sufficient since morphological changes in many cases are detected too late to influence treatment strategies. Therefore, there is an obvious need for markers that could provide a reliable and early assessment of treatment efficacy. In a recent report it was found that metabolic changes reflecting tumor progression may appear earlier than morphological alteration as assessed with MRI [2]. Also in a recent review, Nelson stressed the insufficiency of morphology assessment and called for utilizing metabolic and physiological MR methods to assess therapeutic response [3].

Today MR-spectroscopy (MRS) detecting choline or lactate may provide prognostic information in high-grade astrocytoma and glioblastoma (GBM) [4–7]. In glioma cell lines and rodent tumor models, MRS has shown the potential to provide biomarkers for specific treatments [8–10]. In high-grade gliomas apparent diffusion coefficient (ADC) texture characteristics appear to provide pretreatment prognostic information [11]. However, regarding monitoring of treatment effect in patients using MRI the literature is sparse and without any major breakthrough [12].

In a study on patients with GBM our group has been able to demonstrate that radiotherapy, already after 5 days of 2 Gy fractions, induces significant metabolite pattern changes in the extracellular space of tumor tissue as assessed by microdialysis [13]. We believe that an ideal biomarker should be non-invasive, or at least easy to collect, as well as allow repeated sampling. If a simple blood test could provide the sought for information regarding therapeutic response, that would be optimal from the clinicians’ as well as from the patients’ point of view.

In this report we have analyzed the serum metabolome in the same group of patients and compared the findings in serum to the previously reported metabolite changes of the tumor extracellular compartment following radiotherapy.

## Methods

In the present study untargeted metabolomic profiling using gas-chromatographic- time-of-flight-mass spectroscopy (GC-TOFMS) was performed followed by chemometric bioinformatics analysis in order to identify changes in metabolite patterns in serum samples during radiotherapy. In this way robust and internally validated metabolite patterns consisting of co-varying metabolites, so called latent variables, can be extracted and utilized as specific and predictive biomarkers as well as for facilitated biochemical interpretation.

### Patients, treatment and samples

Eleven patients with radiologic suspicion of a high grade glioma not accessible for surgical resection were included in this study. All patients underwent a stereotactic biopsy to obtain a histopathological diagnosis. After confirmation (10 GBM and one astrocytoma III), microdialysis catheters were implanted with stereotactic technique, one into the contrast-enhancing tumor tissue and one approximately 10 mm outside of the tumor in brain-adjacent to tumor (BAT) [14]. One catheter was put in the abdominal subcutaneous tissue as reference. All catheters had a 10 mm long semi-permeable membrane with a cutoff of 100 kDa (CMA 71, CMA Microdialysis, Stockholm, Sweden). The catheters were perfused with Ringer solution (Perfusion fluid T1; CMA Microdialysis) mixed with Dextran (30 g Dextran 60 1000 mL-1), to prevent microfiltration, and with a flow-rate of 0.3 μl/min (CMA 106 or 107, CMA Microdialysis).

All patients were treated with radiotherapy started within 2 to 5 days after surgery. A standard schedule of 2 Gy × 30 were given to eight of the patients. Three patients in poor general condition were treated using hypofractionationated schedules 3 Gy × 13 for two patients, and 3.4 Gy × 10 for one patient. Fasting serum samples were collected at admittance to the ward, on the same day but before the first treatment session and in the morning after the second and fifth radiotherapy fraction (Table 1). The extracellular compartment were collected every second hour from surgery to the morning after the fifth radiotherapy fraction. Three samples for analysis were selected before the first radiation dose and samples for analysis were matched to the same time-span as for the serum samples. The results from the extracellular compartment in tumor tissue and BAT in the same group of patients have been reported earlier [13].

**Table 1:** Overview of serum samples

Day	−4	0	2	5
Pat. 1	x	x		
Pat. 2	x	x	x	x
Pat. 3	x	x	x	
Pat. 4	x	x	x	x
Pat. 5	x		x	x
Pat. 6	x	x	x	
Pat. 7	x	x	x	x
Pat. 8	x	x	x	x
Pat. 9	x	x	x	x
Pat. 10	x	x	x	x
Pat. 11	x	x		x

Day is given in relation to irradiation treatment. x = sample collected for analysis

All patients participated voluntarily and gave their fully informed consent. The study was approved by the Ethics committee of Umeå University.

### Sample preparation

The serum samples were thawed in room temperature for 30 min before addition 900 μl extraction solution consisting of methanol (90 %) and water (10 %) with 11 internal standards (IS) (7 ng/μl) to 100 μl serum. The samples were extracted using a MM301 vibration Mill (Retsch GmbH & Co. KG, Haan, Germany) for 2 min, 30 Hz, after 2 h on ice, centrifuged 15 min, 4 °C, 14,000 rpm. Then 200 μl of the collected supernatant was transferred to vials and evaporated to complete dryness before methoxymation with 30 μl of methoxyamine solution in pyridine (15 μg/μl) first at 70 °C for 1 h then in room temperature for 16 h. Thereafter, the samples were trimethylsilylated with 30 μl of MSTFA at room temperature for 1 h and before addition of 30 μl of heptane (containing 0.5 μg of methyl stearate).

### GC-TOFMS

GC-TOFMS was used to screen the serum samples for a broad spectrum of metabolites in terms of chemical properties. In this way a robust common metabolite profile can be obtained for all samples and used for further multivariate sample comparisons. Prior to analysis, the samples were randomized and analyzed together with a series of n-alkanes (C_12_-C_32_) to allow retention indexes to be calculated. 1 μl sample was injected splitless by an Agilent 7683 Series autosampler (Agilent, Atlanta, GA) into an Agilent 6980 GC equipped with a 10 m × 0.18 mm i.d. fused-silica capillary column chemically bonded with 0.18 μm DB5-MS stationary phase (J&W Scientific, Folsom, CA). The injector temperature was set at 270 °C. Helium was used as carrier gas at a constant flow rate of 1 ml/min through the column. The purge time was set to 60 s at a purge flow rate of 20 ml/min and an equilibration time of 1 min for every analysis. Initially, the column temperature was kept at 70 °C for 2 min and then increased to 320 °C at 30 °C/min, where it was kept for 2 min. The column effluent was introduced into the ion source of a Pegasus III TOFMS (Leco Corp., St Joseph, MI). The transfer line temperature was set at 250 °C and the ion source temperature at 200 °C. Ions were generated by a 70 eV electron beam at a current of 2.0 mA. Masses were acquired from m/z 50 to 800 at a rate of 30 spectra/s, and the acceleration voltage was turned on after a solvent delay of 165 s. The acquired data was exported to MATLAB 7.11.0 (R2010b) (Mathworks, Natick, MA) as NetCDF files. MATLAB 7.3 (R2006b) (Mathworks, Natick, MA) was used for data processing for the MD samples.

### Hierarchical multivariate curve resolution

Metabolomics screening by means of GC-TOFMS generates a profile of more or less overlapping compounds (metabolites). To be able to obtain a reliable quantification and identification of the detected metabolites for further sample analyses and evaluation the raw GC-TOFMS data has to be resolved into pure chromatographic peaks and mass spectra for each individual metabolite. To achieve this, baseline correction, alignment, time-window settings and hierarchical multivariate curve resolution (HMCR) [15, 16] were performed using in-house developed scripts in Matlab 7.11.0 (R2010b) (Mathworks, Natick, MA). The chromatograms were divided into 56 time windows from which the chromatographic peaks were resolved resulting in a data matrix (**X**) were each row represents one serum sample and each column represents one metabolite. For each metabolite in each sample the area under the chromatographic peak, the relative concentration, is calculated. All peak areas were normalized using the peak areas from the 11 internal standards. To identify the detected compounds the mass spectral profile and retentions index were compared with spectra in an in-house spectra library, the NIST library 2.0 (as of January 31, 2001), and the mass spectra library maintained by the Max Planck Institute in Golm. This was followed by a manual inspection and curation of the data to further resolve co-eluting compounds and to correct for split peaks.

### Data analysis

The pre-treated and HMCR resolved GC-TOFMS data describes the complex interactions taking place in the serum metabolome of GBM patients as a consequence of radiotherapy. To be able to extract information of patterns of co-varying metabolites associated with the effect of radiotherapy methods that can handle variable correlations and allows interpretation of such complex interactions are required. To achieve this, chemometric bioinformatics by means of multivariate projection methods was applied to the processed data in order to detect and evaluate metabolite patterns in serum associated with the effect of radiotherapy. Initially, principal component analysis (PCA) [17] was applied to the data to obtain an overview of the variation in the data and detect possible outliers. Outlier detection was performed in both the PCA model space (scores) as well as in the model residual (distance to model in X (DModX)) and to qualify as an outlier leading to exclusion from the analysis a sample show a clearly deviating pattern in either of these two measures. Furthermore, orthogonal partial least squares-discriminant analysis (OPLS-DA) [18] was used to establish if there was a systematic difference in serum metabolite profile between samples collected before and after treatment.

To be able to further analyze treatment induced changes on an individual patient level, the data was pre-treated with the same approach as in our previous study on the same patients, using the Individual Treatment Over Time (ITOT) normalization [13]. The first two samples from the same patient were categorized as untreated and the last two samples were categorized as treated. The value from the first time point (4 days before treatment) was subtracted from the first two time points (including itself). The original value from time point 2 was then included as the first value in the treated samples and subtracted from itself as well as from time point 3 and 4 resulting in that both the treated and the untreated group were set to have the same starting point, for each patient. Patient one, missing its two last time points, was excluded from the analysis. Patient 5 was also excluded because time point two was missing making it impossible to normalize its treated samples.

The ITOT normalized data was then modeled by OPLS-DA with treatment as the response variable (**y**). OPLS-DA is a supervised projection method that describes the relations between the descriptive data (**X**) and the response variable (**y**) by dividing the systematic variation in **X** in two parts, one related to **y**, predictive and the other unrelated, orthogonal, to **y** [18]. By separating the systematic variation into predictive and orthogonal components the interpretation of the model is greatly facilitated. Variables with low model weight values (|w*| < 0.05), variables unaffected by treatment, were discarded from the modelling.

A *p*-value based on ANOVA on the cross-validated OPLS-DA models was used for model validation. The individual metabolites were tested for significance using a Student’s *t*-test, where *p*-values < 0.05 were considered to be significant. To be able to investigate the whole metabolite pattern in serum compared to the whole metabolite pattern in the extracellular compartment in tumor from our previous study, the cross-validated score values for each model were compared in the same analysis.

## Results

### Patients

All patients did undergo the stereotactic procedure and the following period of microdialysis and radiotherapy without any procedure-related side effects or complications.

### Data processing

From the acquired serum GC-TOFMS data 159 putative metabolite peaks were resolved using HMCR. Of these 58 (36 %) could be identified by means of library comparisons. The unidentified metabolites still retained information regarding retention time index and fragmentation patterns and were thus kept in the analysis. This can be compared to the extracellular compartment from tumor tissue where, 151 metabolites were reliably detected and quantified and 67 out of those metabolites were identified (44 %) [13].

### Differences in metabolic pattern due to treatment

**Fig. 1:**
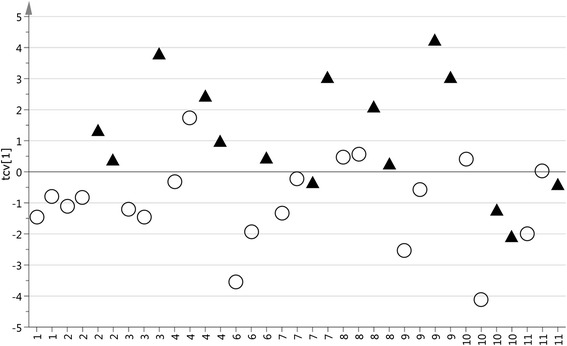
Cross-validate score plot separating samples collected before and during treatment. The x-axis consits of the 10 patients included. The *circles* represent the time points before treatment and the *triangles* represents the time points during treatment. The OPLS-DA model consisted of 1 predicted and 1 orthogonal component and predicted 31.7 % of the response variation (Q2 = 0.317). The *p*-value based on ANOVA on the cross-validated OPLS-DA model is 0.01

**Fig. 2:**
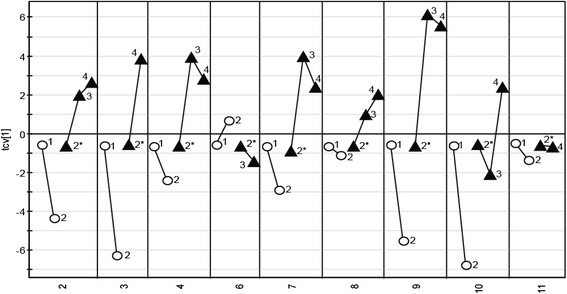
Cross-validated score plot based on metabolites in serum affected by treatment. The x-axis consists of the nine patients included. The *circles* represent the two time points before treatment started and the *triangles* the time points during treatment. The labels correspond to the sampling time point. 2* was calculated and used as a reference point in ITOT normalization of the treated samples. The OPLS-DA model consisted of 1 predicted and 1 orthogonal component and predicted 31.9 % of the response variation (Q2 = 0.319). The *p*-value based on ANOVA on the cross-validated OPLS-DA model is 0.006

No outliers were found in the initial PCA analysis of the HMCR processed data (not shown). As a first modelling step to verify possible treatment specific alterations of the serum metabolite profile OPLS-DA was applied directly on the HMCR processed data. The cross-validated score plot from the calculated OPLS-DA model (Fig. 1) show that there is a general systematic difference in metabolite profile between serum samples taken before and after treatment (*p* = 0.01). To further investigate the patient specific response to treatment the ITOT normalized data was analyzed using OPLS-DA with treatment as the response variable. Based on the model weights, variables were selected and a new OPLS-DA model was calculated. The cross-validated score plot from the final OPLS-DA model (Fig. 2) indicate that the majority of the patients show a metabolic response to radiotherapy in serum (*p* = 0.006). Notably, patients 6 and 11 show a more moderate or no response to the treatment compared to the other patients, indicating a possibility to detect and follow individual differences of treatment in serum close to real-time.

In serum, 84 metabolites passed the cut off criteria (|w*| > 0.05) (Table 2) and 25 of them also showed a significant univariate *p*-value (<0.05). Sixteen metabolites showed higher levels during and after treatment as compared to before treatment while 68 metabolites showed lower levels (Table 2). Out of the identified metabolites, creatinine, threonine, glyceric acid, tyrosine, oleic acid and methionine had the strongest correlation to treatment with lower serum levels after treatment.

**Table 2:** Identified metabolites in serum that were found affected by radiotherapy in patients with GBM

Metabolite	Corr. serum
Aminomalonic acid	↓
Arachidonic acid	↓
Arginine	↓*
Asparagine	↓*
beta-D-Methylglucopyranoside	↓*
Butanoic acid	↓*
Citric acid	↑
Creatinine	↓*
Cysteine	↓
Dehydroascorbic acid dimer	↑
Glutamate	↓
Glutamine	↓*
Glyceric acid	↓*
Glycerol-3-phosphate	↓*
Glycine	↓
Linolenic acid	↓*
Lysine	↓
Methionine	↓*
myo-Inositol	↓
Octadecanoic acid	↓
Oleic/Elaidic acid	↓*
Ornithine	↓*
Phenylalanine	↓
Threonine	↓*
Tryptophan	↓
Tyrosine	↓*
Urea	↓
Valine	↓

Corr. Serum represent the correlation to treatment

↑ denotes increased levels and ↓ denotes decreased levels following treatment

* = *p* < 0.05 calculated with a Student’s *t*-test

The metabolomic findings in extracellular fluid from tumor tissue and BAT from our previous report, in the very same patients, have been included in Additional file 1: Table S1 for comparison. When comparing the results we found metabolites that were affected by treatment in both serum and extracellular fluid from both tumor tissue and BAT. Ornithine, tyrosine and urea decreased in both serum and tumor. Glutamine and glutamate decreased in serum but increased in tumor tissue and BAT. Citric acid increased in serum and BAT while creatinine and glyceric acid decreased in serum samples but showed an increase in BAT.

**Fig. 3:**
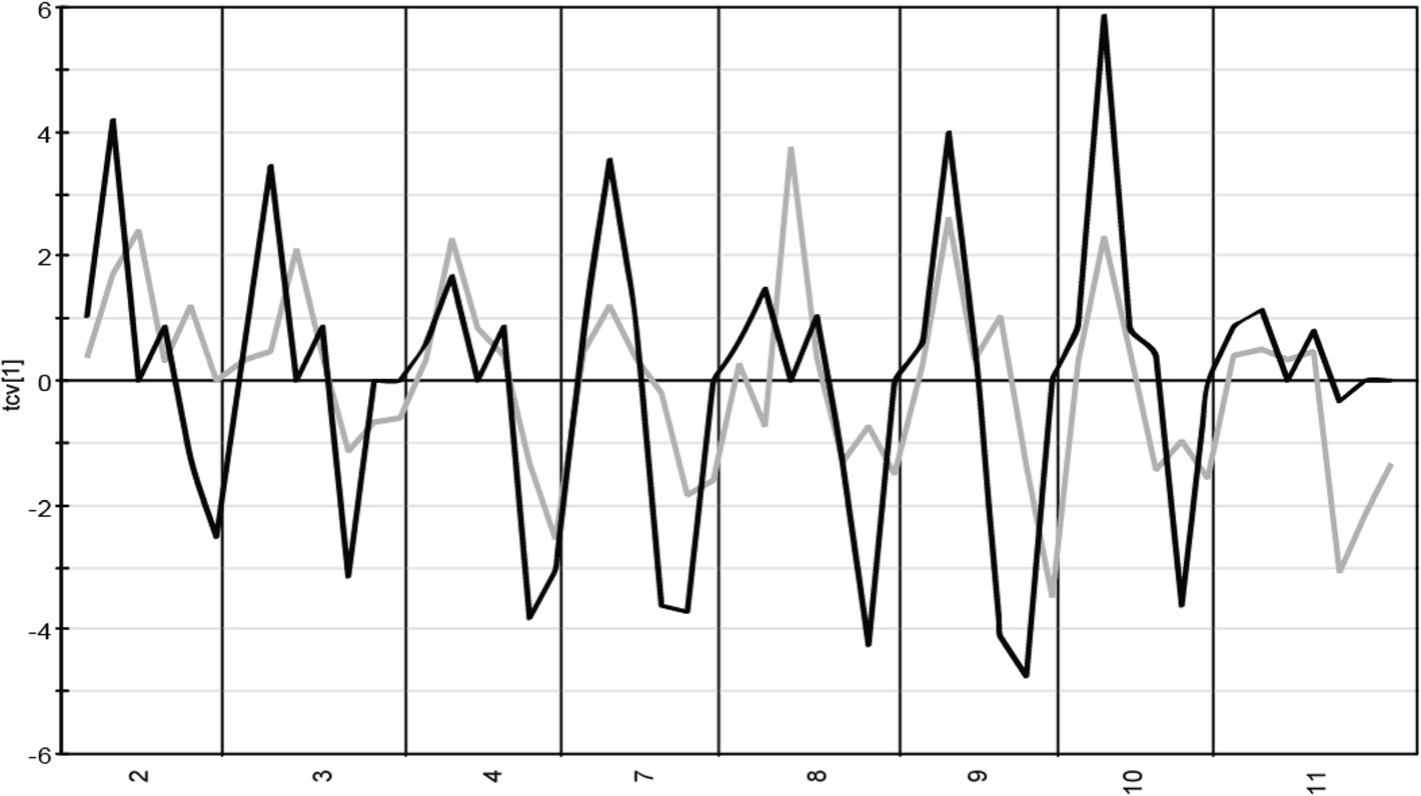
Cross-validated score line plot for serum and extracellular compartment from tumor. The score line plots are based on the final OPLS-DA models from each study. The serum scores is shown by the *black line* and scores from the tumor extracellular compartment is shown by the *grey line*. The x-axis consists of the eight patients that was included in the previous microdialysis study and this study regarding serum samples. The serum OPLS-DA model consisted of 1 predicted and 1 orthogonal component and predicted 31.9 % of the response variation (Q2 = 0.319). The tumor OPLS-DA model consisted of 1 predicted and 1 orthogonal component and predicted 35.5 % of the response variation (Q2 = 0.355). The *p*-value based on ANOVA on the cross-validated OPLS-DA model is 0.006 for the serum model and 0.005 for the tumor model

When interpreting the cross-validated score plots from the modeled data, built on all metabolites that passed the cut off criteria, for both the serum and extracellular compartment from the tumor (Fig. 3) we can see that the pattern before and during treatment is the same or similar for all patients irrespective of compartment. This indicates that the systematic response to treatment in the tumor is reflected in serum even if not all the individual metabolite levels coincide.

## Discussion

This study demonstrates that it is possible to detect metabolic changes in serum during radiotherapy for high grade glioma. We found 84 metabolites that differed between samples collected before treatment compared to samples collected during treatment with radiotherapy. In the end, 28 of those metabolites could be assigned a molecular identity. In a previous study [13], investigating microdialysis samples from the same patients that were included in this study, we demonstrated metabolomic differences correlating with radiation treatment in the extracellular compartment of tumors and brain adjacent to tumor (BAT). In the present study, we have compared the metabolome in serum with previous results from tumor and BAT extracellular compartments. In all three compartments there were significant changes following radiotherapy, indicating that the metabolome may harbor metabolites or patterns of metabolites that can serve as predictive latent biomarkers for radiotherapy treatment of malignant glioma.

We found a response pattern in the serum metabolome similar to the one found earlier in the tumors of the same patients. Therefore it seems like radiotherapy induces systematic changes in the metabolome of different compartments, although the response in terms of individual metabolites may differ between compartments.

Radiotherapy is one of the mainstays for treatment of malignant brain tumors. Modern radiotherapy using three dimensional conformal radiotherapy (3DCRT) or intensity modulated radiotherapy (IMRT) techniques is delivered to the contrast enhancing gross tumor volume (GTV) plus additionally approximately 2 cm margin to control movement and include invasive tumor cells in the surrounding brain tissue. This margin generally includes the BAT region. Thus metabolite changes found in the extracellular compartment from tumor tissue and BAT may reflect actual metabolic events in the tissues. However, one can question if the metabolite changes found in serum reflects changes in the tumor. The present study could not demonstrate a direct correlation between all the metabolite changes in serum and tissue on an individual metabolite level. This may be due to that the observed change in the serum metabolome is only a surrogate marker profile for metabolic events taking place in the tumor. If so, the metabolite profile in serum could be a candidate biomarker for treatment monitoring even if individual serum metabolites lack direct correlation to metabolites in extracellular tissue. Changes in the serum metabolome could also describe radiotherapy induced events in the serum compartment.

In general we found a decrease in metabolite levels in serum during radiotherapy compared to the levels before treatment. Ornithine, tyrosine and urea are decreased in both serum and in the extracellular compartment from tumor during treatment. Glutamine and glutamate on the other hand decreased in serum during treatment while being elevated in the extracellular compartment from tumor.

The decreased serum levels of glutamate, ornithine, arginine and urea point towards a decrease in the urea cycle. Glutamate which is an excitatory neurotransmitter is also involved in growth and development by the regulation of proliferation, survival, migration, and invasion of neuronal progenitors and immature neurons [19]. The decrease in serum glutamate levels could explain the decreased levels of urea, amino acids and fatty acids in serum since glutamate and glutamate metabolism is closely linked to ureagenesis, citric acid cycle, amino acid transferase and lipogenesis among others [20].

Myo-inositol was increased in the extracellular compartment of the tumor and decreased in serum during treatment. The role of myo-inositol is very complex. Myo-inositol is a substrate in the formation of phosphatidylinositol, which in turn is involved in the formation of diacylglycerol and inositol 1,4,5-trisphosphate. Diacylglycerol activates protein kinase C and a cascade of proteolytic enzymes, including matrix metalloproteases which are highly involved in the process of tumor invasion [21]. Inositol trisphosphate stimulates the release of Ca^2+^ making the tumor cells more sensitive to apoptotic stimuli [22]. Inositol is also a precursor for inositol hexaphosphate that, in vitro, has been shown to stimulate apoptosis by upregulating calpain and caspase-3 and downregulate survival factors such as BIRC-2 [23].

We have previously hypothesized that the increase of myo-inositol in tumor tissue after radiation may result from a decrease in the formation of inositol trisphosphate and inositol hexaphosphate [13]. Regardless of the mechanism behind our finding that radiation treatment appears to induce a myo-inositol increase in the tumor and decrease in the serum, it is interesting that previous clinical studies have demonstrated that low levels of myo-inositol in tumor tissue is related to a more aggressive glial tumor [24, 25]. Furthermore, in a previous study we have found that high levels of myo-inositol in the tumor is correlated with longer survival, in both GBM and oligodendroglioma [26]. It would be interesting to investigate whether the increase of myo-inositol in tumor together with a decrease in serum could potentially be a marker for positive treatment effect. In this sense, a larger study will be needed that also incorporates data on clinical response.

We have looked at the individual molecular response from the 11 patients included in the study. As shown in Fig. 2, all patients except for patient 6 and 11 seem to have a similar molecular response to treatment based on the metabolites analyzed in this study. The biological reason for the different metabolite patterns for these two patients is to us not known. Since this study is performed on such a small group of patients reliable statistics regarding the association with survival will not be conclusive. Instead, what this study shows is that different patients have different metabolic starting points and they respond differently to treatment at a metabolite level.

The metabolite alterations detected in this study could arguably be attributed to differences in concurrent medication, perioperative procedures and dietary intake, or they could represent indirect changes due to radiotherapy-induced effects on the hypothalamic-pituitary axis (HPA). However, the perioperative procedures were largely standardized for this study. The patients were mobilized with normal oral nutrition at base-line sampling and during the treatment. In addition, the patients’ blood-glucose levels were monitored and kept below 7 mmol, as it is known that the routinely administered betamethasone affects glucose metabolism. It has also been shown that short term treatment with the steroid prednisolone induces catabolism, resulting in changes in amino acid metabolism and elevation of serum levels of amino acids [27]. However, this is contradictory to our finding of a general decrease in the serum amino acids levels, thus we consider it unlikely that the herein reported metabolite alterations are related to differences in steroid treatment. Furthermore, during the course of the study the patients only received 10–17.5 Gy of radiation, and the planning target volume only in some cases may have partially included the hypothalamus and in no case the pituitary [28, 29]. Because radiation-induced effects of the HPA have only been reported for larger doses, we believe our findings are not related to effects of HPA radiation.

## Conclusion

The results of this study, on a limited series of patients, indicate that serum metabolomics could possibly be a feasible and accessible alternative for assessing response to radiotherapy in malignant glioma. We observed a significant change in metabolite pattern in serum during the early phase of radiotherapy. This change may constitute a possible latent biomarker for treatment outcome and warrants validation in larger prospective cohorts. Correlations of changes on an individual metabolite level between serum and the extracellular compartment was inconclusive. The changes in the serum metabolome may therefore be considered a surrogate biomarker of biological events, though it may not directly reflect changes in irradiated tissue on a molecular level. Further studies are needed to clarify the connections between changes in serum and the extracellular compartment.

## Additional file

Additional file 1: Table S1. Identified metabolites in serum, tumor and BAT that were found affected by radiotherapy. Tumor and BAT samples were collected by microdialysis. (PDF 110 kb)

## References

[1] Stupp R, Hegi ME, Mason WP, van den Bent MJ, Taphoorn MJB, Janzer RC (2009). Effects of radiotherapy with concomitant and adjuvant temozolomide versus radiotherapy alone on survival in glioblastoma in a randomised phase III study: 5-year analysis of the EORTC-NCIC trial. Lancet Oncol.

[2] Hutterer M, Nowosielski M, Putzer D, Waitz D, Tinkhauser G, Kostron H (2011). O-(2-18 F-fluoroethyl)-L-tyrosine PET predicts failure of antiangiogenic treatment in patients with recurrent high-grade glioma. J Nucl Med.

[3] Nelson SJ (2011). Assessment of therapeutic response and treatment planning for brain tumors using metabolic and physiological MRI. NMR Biomed.

[4] Saraswathy S, Crawford FW, Lamborn KR, Pirzkall A, Chang S, Cha S (2009). Evaluation of MR markers that predict survival in patients with newly diagnosed GBM prior to adjuvant therapy. J Neurooncol.

[5] Crawford FW, Khayal IS, McGue C, Saraswathy S, Pirzkall A, Cha S (2009). Relationship of pre-surgery metabolic and physiological MR imaging parameters to survival for patients with untreated GBM. J Neurooncol.

[6] Majos C, Bruna J, Julia-Sape M, Cos M, Camins A, Gil M (2011). Proton MR spectroscopy provides relevant prognostic information in high-grade astrocytomas. AJNR Am J Neuroradiol.

[7] Li X, Jin H, Lu Y, Oh J, Chang S, Nelson SJ (2004). Identification of MRI and 1H MRSI parameters that may predict survival for patients with malignant gliomas. NMR Biomed.

[8] Venkatesh HS, Chaumeil MM, Ward CS, Haas-Kogan DA, James CD, Ronen SM (2012). Reduced phosphocholine and hyperpolarized lactate provide magnetic resonance biomarkers of PI3K/Akt/mTOR inhibition in glioblastoma. Neuro Oncol.

[9] He T, Doblas S, Saunders D, Casteel R, Lerner M, Ritchey JW (2011). Effects of PBN and OKN007 in rodent glioma models assessed by 1H MR spectroscopy. Free Radic Biol Med.

[10] Lemasson B, Christen T, Tizon X, Farion R, Fondraz N, Provent P (2011). Assessment of multiparametric MRI in a human glioma model to monitor cytotoxic and anti-angiogenic drug effects. NMR Biomed.

[11] Brynolfsson P, Nilsson D, Henriksson R, Hauksson J, Karlsson M, Garpebring A et al. ADC texture-An imaging biomarker for high-grade glioma? Med. Phys. 2014;41(10). doi:10.1118/1.4894812. 10.1118/1.4894812 25281955

[12] Liimatainen T, Hakumaki JM, Kauppinen RA, Ala-Korpela M (2009). Monitoring of gliomas in vivo by diffusion MRI and (1)H MRS during gene therapy-induced apoptosis: interrelationships between water diffusion and mobile lipids. NMR Biomed.

[13] Wibom C, Surowiec I, Moren L, Bergstrom P, Johansson M, Antti H (2010). Metabolomic Patterns in Glioblastoma and Changes during Radiotherapy: A Clinical Microdialysis Study. J Proteome Res.

[14] Roslin M, Henriksson R, Bergstrom P, Ungerstedt U, Bergenheim AT (2003). Baseline levels of glucose metabolites, glutamate and glycerol in malignant glioma assessed by stereotactic microdialysis. J Neuro-Oncol.

[15] Jonsson P, Gullberg J, Nordstrom A, Kusano M, Kowalczyk M, Sjostrom M (2004). A strategy for identifying differences in large series of metabolomic samples analyzed by GC/MS. Anal Chem.

[16] Jonsson P, Johansson AI, Gullberg J, Trygg J, J A, Grung B (2005). High-throughput data analysis for detecting and identifying differences between samples in GC/MS-based metabolomic analyses. Anal Chem.

[17] Wold S, Esbensen K, Geladi P (1987). Principal Component Analysis. Chemom Intell Lab Syst.

[18] Trygg J, Wold S (2002). Orthogonal projections to latent structures (O-PLS). J Chemom.

[19] Komuro H, Rakic P (1993). Modulation of neuronal migration by NMDA receptors. Science.

[20] DeBerardinis RJ, Lum JJ, Hatzivassiliou G, Thompson CB (2008). The biology of cancer: Metabolic reprogramming fuels cell growth and proliferation. Cell Metab.

[21] Uhm JH, Dooley NP, Villemure JG, Yong VW (1996). Glioma invasion in vitro: Regulation by matrix metalloprotease-2 and protein kinase C. Clin. Exp. Metastasis.

[22] Szado T, Vanderheyden V, Parys JB, De Smedt H, Rietdorf K, Kotelevets L (2008). Phosphorylation of inositol 1,4,5-trisphosphate receptors by protein kinase B/Akt inhibits Ca2+ release and apoptosis. Proc Natl Acad Sci U S A.

[23] Karmakar S, Banik NL, Ray SK (2007). Molecular mechanism of inositol hexaphosphate-mediated apoptosis in human malignant glioblastoma T98G cells. Neurochem Res.

[24] Castillo M, Smith JK, Kwock L (2000). Correlation of myo-inositol levels and grading of cerebral astrocytomas. Am J Neuroradiol.

[25] Kallenberg K, Bock HC, Helms G, Jung K, Wrede A, Buhk JH (2009). Untreated Glioblastoma Multiforme: Increased Myo-inositol and Glutamine Levels in the Contralateral Cerebral Hemisphere at Proton MR Spectroscopy. Radiology.

[26] Moren L, Bergenheim AT, Ghasimi S, Brannstrom T, Johansson M, Antti H (2015). Metabolomic Screening of Tumor Tissue and Serum in Glioma Patients Reveals Diagnostic and Prognostic Information. Metabolites.

[27] Ellero-Simatos S, Szymanska E, Rullmann T, Dokter WHA, Ramaker R, Berger R (2012). Assessing the metabolic effects of prednisolone in healthy volunteers using urine metabolic profiling. Genome Med..

[28] Darzy KH (2013). Radiation-induced hypopituitarism. Curr. Opin. Endocrinol. Diabetes Obes..

[29] Laughton SJ, Merchant TE, Sklar CA, Kun LE, Fouladi M, Broniscer A (2008). Endocrine outcomes for children with embryonal brain tumors after risk-adapted craniospinal and conformal primary-site irradiation and high-dose chemotherapy with stem-cell rescue on the SJMB-96 trial. J Clin Oncol.

