# Supplementary material for: Characterization of the serum metabolome following radiation treatment in patients with high-grade gliomas
Source: Radiat Oncol. 2016 Apr 2;11:51. doi: 10.1186/s13014-016-0626-6 (PMC4818859; doi:10.1186/s13014-016-0626-6)
Supplement: Additional file 1: Table S1. — Identified metabolites in serum, tumor and BAT that were found affected by radiotherapy. Tumor and BAT samples were collected by microdialysis. (PDF 110 kb) [file 13014_2016_626_MOESM1_ESM.pdf]

Table S1. Identified metabolites in serum, tumor and BAT that were found affected by radiotherapy. Tumor and BAT samples were collected by microdialysis.

Metabolite	Corr. Serum	Corr.Tumor	Corr.BAT
**Essential amino acids**			
Lysine	↓		
Methionine	↓*		
Phenylalanine	↓		
Threonine	↓*		
Valine	↓		
**Amino acids**			
Alanine		↑*	
Aminomalonic acid	↓		
Arginine	↓*		
Asparagine	↓*		
Cystathionine			↑*
Cysteine	↓		
Glutamate	↓	↑	↑
Glutamine	↓*	↑	↑*
Glycine	↓		
Ornithine	↓*	↓*	↓
Pyroglutamic acid			
Tryptophan	↓		
Tyrosine	↓*	↓	
**Alcohols**			
Arabitol			↑*
Ethanolamine		↓	↑*
Glycerol-3-phosphate	↓*		
myo-Inositol	↓	↑*	
Ribitol			
**Fatty Acids**			
Arachidonic acid	↓		
Butanoic acid	↓*		
Linoleci acid	↓*		
Octadecanoic acid	↓	↑*	↓
Oleic/Elaidic acid	↓*		
**Saccarides**			
Arabinose		↓	↑*
Fructose			↑
Galactose		↓	
Glucose			↑
Glucopyranose (glucose?)			↑
Lactulose			↑
beta-D-Methylglucopyranoside	↓*		
Nigerose		↑*	
Palatinose			↑
Trehalose			↑
Xylulose		↑*	
**Citric acid cycle**			
Citric acid	↑		↑*
Succinic acid			↓
**Misc**			
Chlorobenzoic acid		↑	↑
Creatinine	↓*		↑
Dehydroascorbic acid dimer	↑		
Glutaric acid		↑	
Glyceric acid	↓*		↑
Glycerol			↑*
Itaconic acid		↑	↑*
Pentonic acid			↑*
Phosphoric acid			
S-Methyl-L-Cysteine		↓	
Urea	↓	↓	↑

Corr. Serum, Corr.Tumor and Corr.BAT represent the correlation to treatment.
↑ denotes increased levels and ↓ denotes decreased levels following treatment.
* = $p < 0.05$ calculated with a Student's t-test.
